# From Karl Wurm and Guy Scadding's staging to ^18^F-FDG PET/CT scan phenotyping and far beyond: perspective in the evading history of phenotyping in sarcoidosis

**DOI:** 10.3389/fmed.2023.1174518

**Published:** 2023-05-10

**Authors:** Spyros A. Papiris, Lykourgos Kolilekas, Natalia Rivera, Michail Spanos, Guoping Li, Priyanka Gokulnath, Emeli Chatterjee, Alexandros Georgakopoulos, Maria Kallieri, Andriana I. Papaioannou, Thomas Raptakis, Vasiliki Apollonatou, Elvira-Markela Antonogiannaki, Elias Gialafos, Sofia Chatziioannou, Johan Grunewald, Effrosyni D. Manali

**Affiliations:** ^1^2nd Pulmonary Medicine Department, Medical School, General University Hospital Attikon, National and Kapodistrian University of Athens, Athens, Greece; ^2^7th Pulmonary Department, Athens Chest Hospital “Sotiria”, Athens, Greece; ^3^Respiratory Medicine Division, Department of Medicine Solna, Karolinska Institutet, Stockholm, Sweden; ^4^Cardiovascular Research Center, Simches 3 Massachusetts General Hospital, Boston, MA, United States; ^5^2nd Department of Radiology, Nuclear Medicine Section, Medical School, General University Hospital “Attikon”, National and Kapodistrian University of Athens, Athens, Greece; ^6^1st Respiratory Medicine Department, Athens Medical School, Sotiria Chest Hospital of Athens, National and Kapodistrian University of Athens, Athens, Greece; ^7^4th Pulmonary Department, Athens Chest Hospital “Sotiria”, Athens, Greece; ^8^Department of Cardiology, Medical School, General University Hospital “Attikon”, National and Kapodistrian University of Athens, Athens, Greece; ^9^First Department of Neurology, Medical School, Aeginition Hospital, National and Kapodistrian University of Athens, Athens, Greece; ^10^Division of Nuclear Medicine, Biomedical Research Foundation of the Academy of Athens, Athens, Greece

**Keywords:** sarcoidosis, chest roentgenogram staging, ^18^F-FDG PET/CT scan phenotyping, omics, personalized treatment

## Abstract

Sarcoidosis is an inflammatory granulomatous disease of unknown etiology involving any organ or tissue along with any combination of active sites, even the most silent ones clinically. The unpredictable nature of the sites involved in sarcoidosis dictates the highly variable natural history of the disease and the necessity to cluster cases at diagnosis based on clinical and/or imaging common characteristics in an attempt to classify patients based on their more homogeneous phenotypes, possibly with similar clinical behavior, prognosis, outcome, and therefore with therapeutic requirements. In the course of the disease's history, this attempt relates to the availability of a means of detection of the sites involved, from the Karl Wurm and Guy Scadding's chest x-ray staging through the ACCESS, the WASOG Sarcoidosis Organ Assessment Instruments, and the GenPhenReSa study to the ^18^F-FDG PET/CT scan phenotyping and far beyond to new technologies and/or the current “omics.” The hybrid molecular imaging of the ^18^F-FDG PET/CT scan, by unveiling the glucose metabolism of inflammatory cells, can identify high sensitivity inflammatory active granulomas, the hallmark of sarcoidosis—even in clinically and physiologically silent sites—and, as recently shown, is successful in identifying an unexpected ordered stratification into four phenotypes: (I) hilar–mediastinal nodal, (II) lungs and hilar–mediastinal nodal, (III) an extended nodal supraclavicular, thoracic, abdominal, inguinal, and (IV) all the above in addition to systemic organs and tissues, which is therefore the ideal phenotyping instrument. During the “omics era,” studies could provide significant, distinct, and exclusive insights into sarcoidosis phenotypes linking clinical, laboratory, imaging, and histologic characteristics with molecular signatures. In this context, the personalization of treatment for sarcoidosis patients might have reached its goal.

## Introduction

Sarcoidosis is a systemic inflammatory disease of unknown etiology, which occurs in populations worldwide and involves the lungs and the intrathoracic lymph nodes (1–3). The etiologically implicated antigen “still eludes us,” and the disease is considered a dysregulated, immune-mediated response due to its presence, persistence, and failure to clear, leading to tissue granulomas formation (4, 5). The histological hallmark of the disease indeed constitutes well-formed, non-caseating granulomas that may localize in any organ or tissue without boundaries and with any combination patterns of active sites of involvement in most of the cases, notably, even in the clinically silent (6, 7) cases. The detection of granulomas with the abovementioned characteristics is never pathognomonic for the disease (8). Therefore, it is necessary to ensure diagnosis in most cases is associated with the combination of compatible clinical, laboratory, and imaging characteristic patterns with histologic findings, as well as the exclusion of any other etiology of granulomatous inflammation (1, 9).

However, clinical compatibility for sarcoidosis in most cases—beyond the well-known and quite pathognomonic clinical syndromes (phenotypes), such as Löfgren's syndrome and Heerfordt's syndrome—constitutes primarily the unpredictable and “anarchic” tissue distribution of the disease in combination with some characteristic imaging features from the lungs, in addition to the asymptomatic or oligosymptomatic disease's first appearance (1, 10–12). Due to the unpredictability of the sites involved in sarcoidosis, the disease has a highly variable natural history, and since every sarcoidosis patient represents a distinctive case of theirown, individual management strategies may be imperative (13–20).

Clustering or better phenotyping patients with the disease at diagnosis on the basis of clinical and/or imaging common characteristics is an old attempt to assign patients with an unpredictable disease to more or less homogeneous groups possibly with similar clinical behavior, prognosis, and outcome and therefore with similar therapeutics requirements (21–30). In the course of the history of the disease, this attempt relates to the availability of the current means of detection of any site of involvement in a systemic disease, from the chest x-ray of Karl Wurm and Guy Scadding's staging and through the ACCESS study, the WASOG Sarcoidosis Organ Assessment Instrument, and the GenPhenReSa study to the ^18^F-fluoro-2-deoxyglucose (^18^F-FDG) positron emission tomography (PET) computed tomography (CT) scan phenotyping and far beyond to new technologies and/or the current “omics” availability (31–41). Stratification of sarcoidosis patients based on T-cell count in Bronchoalveolar Lavage (BAL) and gallium scan, the presence of impaired physiology, fibrosis, and pulmonary hypertension, as well as clinical activity, as reflected by acute or non-acute disease in onset, treatment, and long-term treatment requirements, and the clinical outcome status of the disease have also been described (24, 42–48). Successful phenotyping, to be clinically useful by the physicians in everyday clinical practice, should be easy, simple, reliable in unraveling most, if not all, sites of disease involvement, less expensive, reproducible worldwide in different populations with the disease, and able to offer information concerning the clinical behavior of the individual phenotypes, thus providing valuable information for the decision-making process (49). The corpus of this research article has been focused on the initial evaluation of the sarcoidosis patient.

## Ordering the unpredictable

Sometimes sarcoidosis creates order within itself. Sven Löfgren was the first to associate erythema nodosum and bilateral hilar lymphadenopathy with sarcoidosis—an early or acute manifestation of the disease (fever and polyarthritis commonly coexist) that is fairly distinct from tuberculosis, with good prognosis—an assumption that maintains its value till present (50–52). Cristian Heerfordt was the first to describe that, in sarcoidosis, the “febris uveoparotidea subchronica” (fever, parotid enlargement, and uveitis) was occasionally associated with the seventh nerve palsy and other rare manifestations of the disease (53). Both the above two phenotypes though fairly uncommon maintain a significant diagnostic value not necessitating histologic confirmation in most if not in all patients. Prognosis is excellent in Löfgren's, and indeterminate in the Heerfordt's syndrome (1, 8). However, despite the fact that the abovementioned phenotypes are narrow in terms of clinical manifestations and are relatively homogeneous, nothing is known about other silent systemic sites of active disease; better detection and definitive identification of these silent systemic sites are possible with the application of new technologies (7). As disease evolved, technology and research followed its lead.

## In the beginning it was Wilhelm Conrad Röntgen

In the beginning, it was Wilhelm Conrad Röntgen (recipient of Nobel Prize in Physics in 1901) who was unaware of sarcoidosis, and by discovering the electromagnetic radiation known as Röntgen rays (X-rays), he offered the availability of chest roentgenogram to the pioneers of the disease (Ernest Besnier, Caesar Boeck, and Jörgen Schaumann, who were the men behind the sarcoidosis disease and the disease was named after them in early days) rendering them aware that the skin, eyes and joints disease in front of them was part of an internal organ, systemic disease involving almost always the lungs and the intrathoracic lymph nodes: the indispensable first encounter of every physician with any sarcoidosis patient to this day (54–56).

## Karl Wurm and Guy Scadding's staging first step through chest radiology

**Figure 1:**
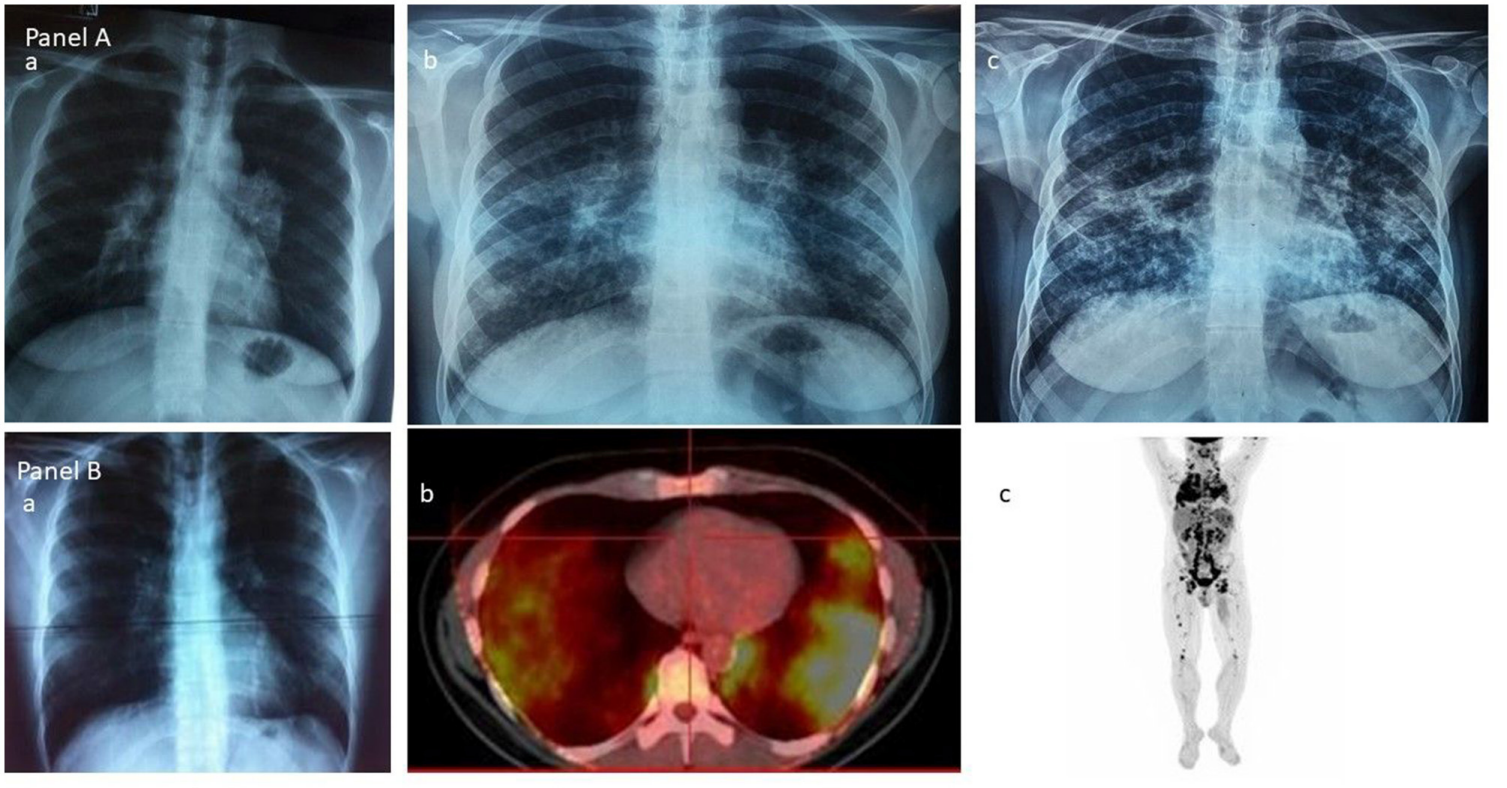
**(A)** The Scadding's vision of sarcoidosis through the chest roentgenogram: group 1—enlarged hilar lymph-nodes; group 2—hilar nodes and lung shadowing; and group 3—lung shadowing. **(B)** The current vision of sarcoidosis through the ^18^F-FDG PET/CT scan: (a) and (b) the lungs; (c) the whole body.

The first chest röentgenogram (x-rays) classification of sarcoidosis, which was designed by Karl Wrum, was divided into three stages (stage I bilateral hilar–mediastinal lymphadenopathy, stage II lungs reversible involvement and stage III lungs fibrosis) and was the first attempt to draw prognostic considerations of the disease, and accordingly arriving at therapeutic decisions for sarcoidosis patients (31, 32). It belongs to Guy Scadding the current chest radiological staging of sarcoidosis in “four groups” as firstly reported, [group 1) enlarged hilar lymph-nodes, group 2) hilar nodes and lung shadowing, group 3) lung shadowing and group 4) fibrosis, though not always “sharply demarcated”] and belong to him the seminal conclusions regarding the prognostic significance of the above radiologic grouping as well as the fundamental clinical advice that corticosteroids are necessary only in a minority of patients (33) (Figure 1A), all of which were determined by Guy Scadding. Moreover, Guy Scadding also confirmed previous observations reporting the benign clinical course and spontaneous resolution of the Löfgren's syndrome phenotype. Several years later, Johan Grunewald and Anders Eklund provided additional significant information regarding the influence of the genetic background, human leukocytes antigen (HLA) polymorphisms, particularly the HLA-DRB1^*^03 allele on the outcome of this phenotype—a new step in the effort to draw information about phenotypes from the science advancement adding to the clinical information the results of the “bench” (11, 23, 57–61). During the early Scadding's times, the awareness of extrathoracic manifestations of sarcoidosis was mostly limited to the skin, eyes, parotids, superficial lymph nodes, joints, and few others, and there were no reliable conclusions that could be added regarding the clinicians' influence on disease's clinical behavior, prognosis, and outcome. In modern times, the necessity to evolve from Karl Wurm and Guy Scadding's staging was unavoidable and possible because of the advancement of technology and scientific thinking (49).

## The ACCESS study for the definition of organ involvement in sarcoidosis

To solve the problem of the elusive identification of the etiologically implicated antigen in sarcoidosis, the National Heart, Lung, and Blood Institute (NHLBI) set up the A Case Control Etiologic Study of Sarcoidosis (ACCESS) multicenter study (34–36). In present times, the ACCESS study proposed an instrument to assess and define organ involvement in sarcoidosis, a clear step that has been taken ahead to evolve from Karl Wurm and Guy Scadding's staging. It was successful in identifying differences in HLA gene associations with sarcoidosis among European and African Americans; however, for several reasons, the instrument failed to address all possible sites of the disease's activity, which is indispensable to identifying phenotypes (62). Furthermore, the development of new technologies made the ACCESS instrument outdated, and the necessity for organ assessment in sarcoidosis resulted in developing new instruments.

## The WASOG Sarcoidosis Organ Assessment Instrument

The WASOG Sarcoidosis Organ Assessment Instrument was developed as an update of the previous ACCESS tool to establish reliable criteria for the probability of any organ being involved in the disease in a sarcoidosis patient, in the new technology era. The probability of organ involvement from sarcoidosis was based on two criteria: granulomatous inflammation and compatible clinical manifestation, excluding in both cases alternative etiologies. Based on the above findings, clinical manifestations were graded as (1) highly probable, 90% likelihood, (2) probable, 50–90% likelihood, and (3) possible, <50% likelihood. For each manifestation, an agreement of 70% was needed for consensus (Delphi study methodology). Indeterminate probability was defined when consensus was not reached (37).

## GenPhenReSa consortium

The GenPhenReSa consortium is the first attempt, entirely utilizing the WASOG Sarcoidosis Organ Assessment Instrument to identify almost all sites of involvement by the disease, in order firstly to identify reliable and homogeneous phenotypes of sarcoidosis appearance, useful cohorts for further biomedical studies and secondly to attempt possible genotype–phenotype associations were detected. The results of the phenotype module of the GenPhenReSa as evidenced by the cluster analysis of 2,163 Caucasian patients phenotyped in 31 different sarcoidosis expert study centers regard the identification of five “distinct” subgroups identified from organ involvement: (1) abdominal organ involvement, (2) ocular-cardiac-cutaneous-central nervous system disease involvement, (3) musculoskeletal-cutaneous involvement, (4) pulmonary and intrathoracic lymph node involvement, and (5) extrapulmonary involvement, defined by the authors as “homogeneous cohorts useful for further biomedical studies” (38). However, some concerns arise at first glance regarding distinctness and homogeneity since the same authors acknowledge that “new technologies will enable better detection of organ involvement”; therefore, frequencies and clusters of organ involvement are likely to change over time. Indeed, using the abovementioned system to define organ involvement appears laborious in terms of the necessity for several tests and examinations as well as somewhat controversial in terms of the homogeneity of clusters since phenotyping appears to overlap and is somewhat ambiguous.

## ^18^F-FDG PET/CT scan phenotyping

**Figure 2:**
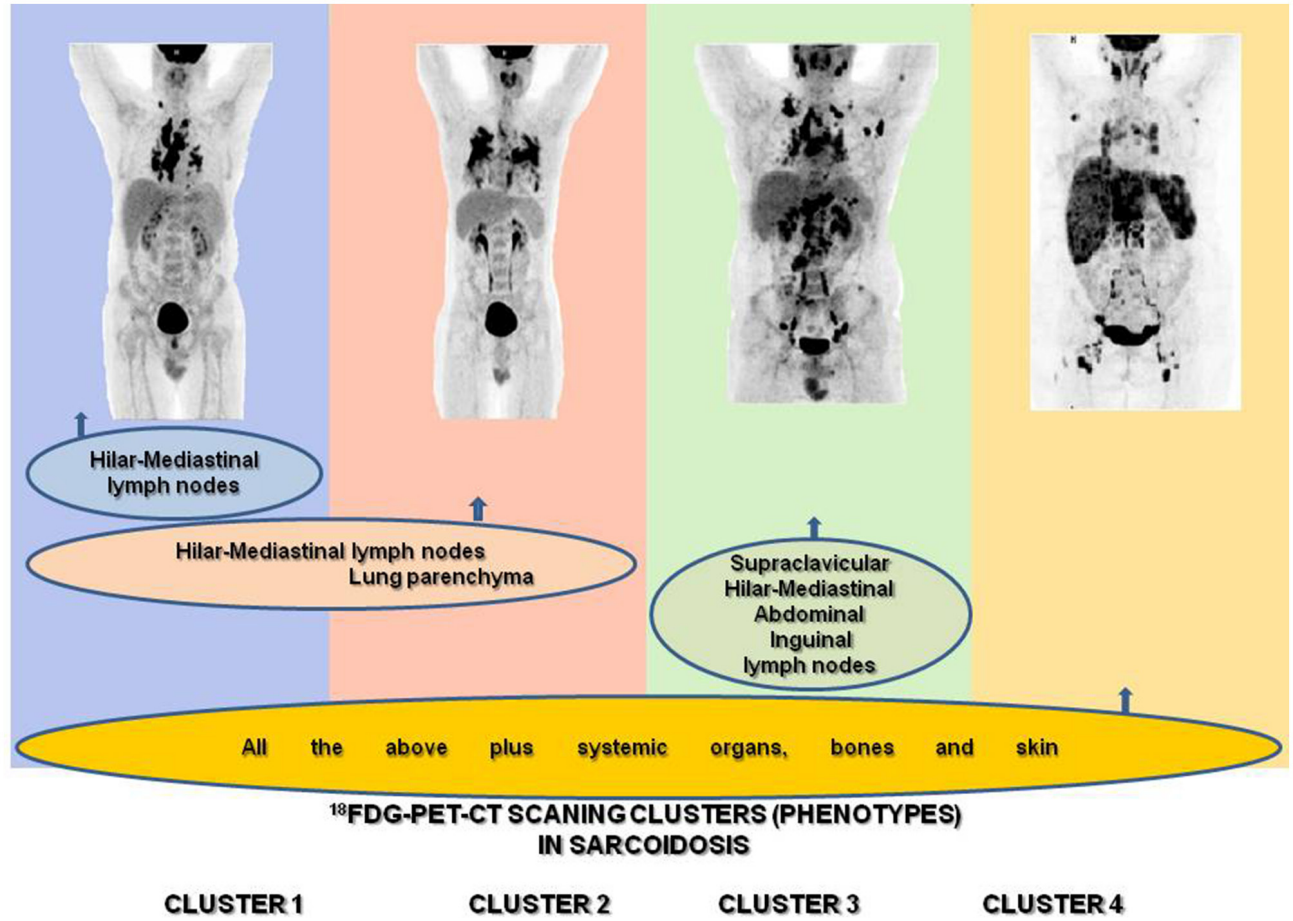
Identification of sarcoidosis ^18^F-FDG PET/CT scan phenotypes according to organ involvement using two-step cluster analysis: (1) hilar–mediastinal nodal, (2) lungs and hilar–mediastinal nodal, (3) an extended nodal supraclavicular, thoracic, abdominal, inguinal, and (4) all the above in addition to systemic organs and tissues such as muscles–bones–spleen and skin.

The hybrid molecular imaging of the ^18^F-FDG PET/CT scan by unveiling glucose metabolism of inflammatory cells is able to identify, with high sensitivity, inflammatory active granulomas, the hallmark of sarcoidosis, even in clinically and physiologically silent sites, providing simultaneously whole-body PET and CT images (63, 64). Therefore, the ^18^F-FDG PET/CT scan appears to be the ideal instrument for the assessment of organs involved in sarcoidosis in detecting their intrinsic inflammatory activity (65–67) (Figure 1B) in the most beneficial fashion. Through the ^18^F-FDG PET/CT scan, investigators may attain a far better understanding of sarcoidosis physical history, behavior, unveiling patterns of disease expression, subdividing patients by clusters, and identifying phenotypes. Recently, Papiris et al. by implementing an ^18^F-FDG PET/CT scan in newly diagnosed, especially in the treatment-naïve patients with sarcoidosis, identified, despite the random distribution of the disease, by a statistical method called hierarchical cluster analysis, an unexpected ordered stratification into four phenotypes: (I) hilar–mediastinal nodal, (II) lungs and hilar–mediastinal nodal, (III) an extended nodal supraclavicular, thoracic, abdominal, inguinal, and (IV) all the above in addition to systemic organs and tissues such as muscles–bones–spleen and skin (39) (Figure 2). Although this approach represents a simplified assessment of sarcoidosis where the use of only one investigative tool and in “one shot” depicts the universal expression of the disease by its active presence, the approach indisputably offered a much logical picture of the disease much closer to the perception of caring clinicians. Furthermore, this “one-shop-stop” in the acquisition of data offers “an open book” approach to the clinicians in getting themselves exposed to every single “page written by the disease” by scientists and experts who have in-depth knowledge and to decide on therapeutic requirements. The organ clusters (phenotypes) identified in this analysis remain far better than anyone else's analysis carried out before and are in accordance with the existing knowledge of sarcoidosis. The phenotypes I and II appeared to coincide with the familial Scadding's first two stages; however, by applying ^18^F-FDG PET/CT scan, all other sites of disease involvement were surely excluded, a sort of “pure organs Scadding's stages I and II,” while in Scadding's staging the other coexisting sites of disease involvement slowly got away from the disease scenario and went unidentified. The phenotype III disclosed was rather suspected in sarcoidosis patients and occasionally observed clinically and by other means such as computerized tomography (CT) scans and echocardiogram (ECHO). However, for the first time, the disease involvement was clearly identified in all possible extensions of the actual site, and a lot of work must be done to identify its prognostic significance and therapeutic requirements (apparently necessitating none). Finally, the phenotype IV appears familial to the clinician-caring sarcoidosis patients since it appears to be satisfying to the current knowledge of the systemic nature of the disease, probably relating to the systemic spreading, from the lungs and hilar–mediastinal lymph nodes to the whole body, of the etiologically implicated antigen. Therefore, by clustering investigations of sarcoidosis through the ^18^F-FDG PET/CT scan, clinically logical phenotypes were identified. However, like all other clustering investigations, some patients may not respect the boundaries but also Scadding's staging by the simplest of the means of investigation, the chest roentgenogram, which clarifies that stages may not be “sharply demarcated,” and we feel right to confirm his elaboration concerning clustering. Based on hierarchical cluster analysis of the study population and implementing adequate preparation with low carbohydrate diet for 24 h followed by 18-h fasting to suppress radiotracer's uptake by normal myocardial cell, no significant difference was detected between the clusters regarding myocardial involvement, suggesting that heart disease could be detected and should be evaluated in any cluster. Given the important prognostic and treatment implications of cardiac involvement, the role of ^18^F-FDG PET/CT scan has been examined extensively. Although this modality requires a specific protocol including diet restriction and being more prone than cardiac magnetic resonance (CMR) to provide false positive results, it is a technique much more sensitive than transthoracic echocardiogram (70 vs. 25%) that should be used as complementary to CMR for the detection of myocardial inflammation and fibrosis, respectively (16, 39, 68, 69). Certainly, the low specificity of ^18^F-FDG PET/CT scan poses several concerns regarding the differentiation with any neoplasm or other inflammatory etiology, but in the patient diagnosed with sarcoidosis, further approach in unusual or suspected sites investigated by additional investigative tools including biopsy should be warranted (70). The ^18^F-FDG PET/CT scan molecular imaging by its high sensibility in detecting organs' intrinsic inflammatory activity, its worldwide availability, the “relatively low” radiation impact, the reasonable cost, and its reliability to evaluate treatment response appears the ideal instrument for phenotyping in sarcoidosis. However, the ^18^F-FDG PET/CT is unable to “see” the eye; therefore, to obtain a complete phenotype, an ophthalmologic examination is mandatory (71–77). Neurological and endocrinology evaluations are also indispensable for thoroughness to detect neurosarcoidosis, small-fiber neuropathy, and abnormal calcium metabolism (78–81). Until further well-designed multicenter studies are performed to confirm the abovementioned findings as universal patterns of disease behavior, we do not consider this study an ideal destination for sarcoidosis and its assessment but rather treat it as just another journey ahead. ^18^F-FDG PET/CT should be used rationally and wisely, its radiation exposure that is comparable to whole-body diagnostic CT should also be taken into consideration and should be balanced by its higher sensitivity for both thoracic and extrathoracic disease. The unveiling of the abovementioned phenotypes, especially in treatment naïve patients at the first glance on diagnosis, appears useful in designing future studies with more homogeneous cohorts, toward the acquisition in sarcoidosis patients of a more personalized medicine approach (39). Currently, relevant studies are lacking; whether ^18^F-FDG PET/CT should be recommended to all patients and whether ^18^F-FDG PET/CT phenotyping could provide additional information on outcome, treatment indications, silent lung disease, such as asymptomatic radiographic stage I included, should be further validated.

## The “omics era”

“Omics” is a high-throughput technology that allows for the comprehensive profiling of various biochemical molecules in a context-dependent manner in multiple organisms. “Omics” studies include genetics, epigenetics, transcriptomics, proteomics, lipidomics, metabolomics, and microbiomics. Given that sarcoidosis is a complex, polygenic disease of unknown cause with diverse clinical phenotypes, the “omics” approach adds another layer of information by delving into the molecular signatures that could provide important insights on the pathogenesis of this disease (4, 5, 41, 82–88). Genetic predisposition and immune dysregulation, inherent to sarcoidosis, are investigated by characterizing both previously known and newly discovered immune-cell-specific pathways, gene expression, and epigenetic modifications that may differ between specific tissues and compartments as well as between progressive and non-progressive disease manifestations (41, 89–94). Through “omics,” we have currently gained access to a very advanced group of techniques that can provide conceptual, mechanistic, and molecular interpretations of phenotypes irrespective of treatment. For instance, genome-wide association studies (GWAS) examined the genetic composition of many genomes to identify variants associated with specific traits or diseases. GWASs provide insight into phenotypic biology, predict clinical outcomes, and reveal causal relationships between risk factors and health outcomes. Recent GWAS in European, African American, and Asian populations identified a non-synonymous single-nucleotide polymorphism (SNP), rs1049550, within the annexin A11 (ANXA11) gene as being associated with susceptibility to sarcoidosis (95). The RNA-sequencing (RNA-seq) technologies, such as bulk and single-cell RNA-sequencing (scRNA-seq), permit unbiased interrogation of the whole transcriptome and deep immunophenotyping of heterogeneous single cells suspensions, respectively. The RNA-seq data analysis for differential gene expression has become a standard method for comparing gene expression among healthy and diseased individuals, tissues, and cell types and can provide valuable information about dysregulated pathways and inciting disease mechanisms. ScRNA-seq can then be combined with spatial transcriptomics and cellular proteomics to provide a “transcriptometabolomic” map of the cell's active status in healthy and diseased conditions. Using this approach, a recent study examined the immunostructural stoichiometry of sarcoidosis patients' granulomas, revealing that granulomas hijack the transcriptional programs that regulate normal lymphoid organ development and alter cytokine and chemokine pathways (96). These findings may explain the increased inflammatory activity and 18F-FDG uptake observed in those tissues and aid in identifying potentially targetable molecules for therapeutic development. Another method that combines single-cell transcriptomics with proteomics, which is called cellular indexing of transcriptomes and epitopes (CITE-Seq), is a sequencing-based method that allows simultaneous quantification of cell surface protein and single-cell sequencing data, enabling both surface marker and transcriptomic phenotyping of immune cells that are dysregulated in various diseases (97). Since cell surface proteins are indicators of cell phenotype and functional status, CITE-seq may provide a superior method for profiling immune cells in sarcoidosis when compared with scRNA-seq or proteomic analysis alone (98). While “omics” cannot be directly applied to everyday clinical practice, they could provide significant, distinct, and exclusive insights into the described phenotypes of sarcoidosis by combining clinical, laboratory, imaging, and histologic characteristics with molecular signatures in a sort of “reverse phenotyping” approach. Eventually, the future may see an algorithm combining all the aforementioned methods to efficiently diagnose, phenotype, and eventually lead to efficient sarcoidosis treatment. However, larger studies need to be conducted to arrive to robust conclusions that can be developed further into clinical applications.

## Perspective in the evading history of phenotyping in sarcoidosis

### Discussion

As we critically reviewed the history of phenotyping in sarcoidosis, we realized that we have constantly been making progress from what could initially be observed by the inquiring minds of scientists only through the naked eye to what we are currently able to observe using the recent great advances in technology, and the progress seems enormous (99–101). For example, the contribution of ^18^F-FDG PET/CT scan in enriching our ability to screen patients with sarcoidosis for all organs and sites involved, even the most silent ones, in “one shot” is indisputable as well as the ability of -omics studies to provide unbiased insights about pathophysiological and molecular signatures that could be representative of specific sarcoidosis phenotypes, is still unperceivable to clinical observation (39, 41). However, to this day, clinical observation and judgment are the first means of capturing and validating the existence of the phenotypes associated with sarcoidosis. Defining and evolving disease phenotypes is perpetually motivated by the desire to better understand the disease and its many obscure aspects (4). The utility of phenotypes becomes increasingly relevant as studies that intend to elucidate disease mechanisms underlying sarcoidosis pathogenesis and to identify biomarkers that can be used for diagnosis, prediction of outcomes, and optimization of disease management can only be carried out in well-phenotyped populations of multiple ethnic origins; therefore, an initiative requiring international collaboration efforts, such as the Multi-Ethnic Sarcoidosis Genomics Consortium (MESARGEN), may bring together many scientists and clinicians from around the world toward this goal. ^1^

Due to its high sensitivity in detecting intrinsic inflammatory activity, ^18^F-FDG PET/CT scanning appears to be the most appropriate tool for guiding and orchestrating our efforts in phenotyping in sarcoidosis in the future. In the era of “omics”, the research could provide unique insights into sarcoidosis phenotypes through the association of clinical, laboratory, imaging, and histologic characteristics with molecular signatures. In this context, the personalization of treatment for sarcoidosis patients might have reached its goal.

## Data availability statement

The original contributions presented in the study are included in the article/supplementary material, further inquiries can be directed to the corresponding author.

## Author contributions

SP: concept and design of the study (text and figures), analysis and interpretation of all data, and wrote the manuscript. LK, NR, MS, GL, PG, and EC: interpretation of the data, wrote parts of the manuscript, and revised the work critically for very important intellectual content. AG and MK: produced the figures and revised the work critically for important intellectual content. AP, TR, VA, E-MA, EG, SC, and JG: major contribution in the interpretation of the data and revised the work critically for important intellectual content. EM: major contribution to the concept of the study, to the acquisition, analysis and interpretation of data, had access to all data, supervised the accuracy and integrity of any part of the work, and wrote the final version of the manuscript with SP. All authors read and approved the final version of the manuscript.

## Footnotes

^1^Available online at: https://mesargen.org/.
